# Neurofibrillary Tangles and the Deposition of a Beta Amyloid Peptide with a Novel N-Terminal Epitope in the Brains of Wild Tsushima Leopard Cats

**DOI:** 10.1371/journal.pone.0046452

**Published:** 2012-10-03

**Authors:** James K. Chambers, Kazuyuki Uchida, Tomoyuki Harada, Masaya Tsuboi, Masumi Sato, Masahito Kubo, Hiroaki Kawaguchi, Noriaki Miyoshi, Hajime Tsujimoto, Hiroyuki Nakayama

**Affiliations:** 1 Department of Veterinary Pathology, Graduate School of Agricultural and Life Sciences, the University of Tokyo, Tokyo, Japan; 2 National Institute of Animal Health, Ibaraki, Japan; 3 Laboratory of Veterinary Pathology, Joint Faculty of Veterinary Medicine, Yamaguchi University, Yamaguchi, Japan; 4 Laboratory of Veterinary Histopathology, Joint Faculty of Veterinary Medicine, Kagoshima University, Kagoshima, Japan; 5 Department of Veterinary Internal Medicine, Graduate School of Agricultural and Life Sciences, the University of Tokyo, Tokyo, Japan; University of South Florida Alzheimer’s Institute, United States of America

## Abstract

Beta amyloid (Aβ) deposits are seen in aged individuals in many of the mammalian species that possess the same Aβ amino acid sequence as humans. Conversely, neurofibrillary tangles (NFT), the other hallmark lesion of Alzheimer’s disease (AD), are extremely rare in these animals. We detected Aβ deposits in the brains of Tsushima leopard cats (*Prionailurus bengalensis euptilurus*) that live exclusively on Tsushima Island, Japan. Aβ42 was deposited in a granular pattern in the neuropil of the pyramidal cell layer, but did not form argyrophilic senile plaques. These Aβ deposits were not immunolabeled with antibodies to the N-terminal of human Aβ. Sequence analysis of the amyloid precursor protein revealed an amino acid substitution at the 7th residue of the Aβ peptide. In a comparison with other mammalian animals that do develop argyrophilic senile plaques, we concluded that the alternative Aβ amino acid sequence displayed by leopard cats is likely to be related to its distinctive deposition pattern. Interestingly, most of the animals with these Aβ deposits also developed NFTs. The distributions of hyperphosphorylated tau-positive cells and the two major isoforms of aggregated tau proteins were quite similar to those seen in Alzheimer’s disease. In addition, the unphosphorylated form of GSK-3β colocalized with hyperphosphorylated tau within the affected neurons. In conclusion, this animal species develops AD-type NFTs without argyrophilic senile plaques.

## Introduction

Neurofibrillary tangles (NFT), one of the diagnostic lesions of Alzheimer’s disease (AD), are rarely found in non-human animal brains. Although the etiology of AD is yet to be elucidated, the “amyloid hypothesis” is widely accepted to explain its pathogenesis [1]. According to this hypothesis, the age-dependent accumulation of beta amyloid (Aβ) peptides in the brain induces a subsequent cascade that culminates in NFT formation. Argyrophilic aggregates of Aβ peptide are called senile plaques, which are another diagnostic lesion of AD.

The AD-related alterations that occur in the brains of animals such as monkeys and dogs have been well studied [2], [3], [4], [5], [6]. However, although these animals frequently form senile plaques with aging, they rarely develop NFT [7], [8], [9]. Even in the few reported animal cases of NFT, no pathological examinations were performed to exclude other diseases that could have caused the NFT to develop [10], [11]. Therefore, it has been a major interest whether AD is a human-specific disease [12], [13].

One of the authors (JKC) has previously reported the occurrence of NFT in the brains of captive cheetahs (*Acinonyx jubatus*) [11]. Subsequently, we have detected NFT and Aβ deposits in the brains of wild Tsushima leopard cats (*Prionailurus bengalensis euptilurus*). According to the phylogenetic tree of living cat species (Felidae), these two species belong to two closely related lineages that diverged approximately 6.7 million years ago [14]. The NFT of the leopard cats were consistent with the pathological characteristics of human AD and were also accompanied by diffuse granular Aβ42 deposits. Interestingly, unlike other animals such as monkeys and dogs [15], aged cheetahs and leopard cats do not develop argyrophilic senile plaques despite the fact that they develop diffuse Aβ deposits in their brains. In the present study, analysis of the leopard cat APP gene detected a base substitution, which altered the N-terminal amino acid sequence of the Aβ protein. Interestingly, many higher mammals that develop argyrophilic plaques, including dogs and monkeys, possess the same Aβ amino acid sequence as humans [16], [17], [18]. The present study provides biological insights into the pathogenesis of AD.

**Table 1:** Immunohistochemical scoring of Aβ42 and AT8.

Case No.	Aβ42	AT8	Sex	Age
1	–	–	M^a^	3-days-old
2	–	–	F^b^	3-years-old
3	–	–	F	Adult^c^
4	–	–	M	Adult
5	–	–	M	Adult
6	–	–	F	Adult
7	–	–	M	Adult
8	–	–	F	Adult
9	++	–	F	Adult
10	+	+	F	Adult
11	++	+	M	Adult
12	+++	++	F	Adult
13	+++	+++	F	Captive for 10 years
14	+++	+++	M	Captive for 15 years

Aβ42 deposition, −: none, +: diffuse deposition in the cerebral cortex,

++: diffuse deposition in the cerebral cortex and hippocampus,

+++: additional speckled deposits. NFT, −: none, +:a few AT8-positive cells were found in the parahippocampal gyrus, ++: AT8-positive cells were found in the parahippocampal gyrus and hippocampal CA1 region,

+++: in addition to the parahippocampal gyrus and hippocampal CA1 region, AT8-positive cells were also found in the ectosylvian gyrus and hippocampal CA3 region.

a Male;

b Female;

c Age unknown.

**Table 2:** Primary antibodies used in the present study.

Specificity	Clone	Dilution	Antigen retrieval	Manufacturer
Aβ x-42	Mouse mono (12F4)	1∶1000	Formic acid	Millipore, Temecula, CA, USA
Aβ N1	Rabbit poly	1∶100	Formic acid	IBL, Gunma, Japan
Aβ pN3	Rabbit poly	1∶100	Formic acid	IBL, Gunma, Japan
Aβ (pan Aβ )	Rabbit poly	1∶100	Formic acid	Chemicon, Temecula, CA, USA
Hyperphosphorylated tau (Ser202/Thr205)	Mouse mono (AT8)	1∶100	Autoclaving	Thermo Scientific, Rockford, IL, USA
Hyperphosphorylated tau (Ser212/Thr214)	Mouse mono (AT100)	1∶100	Autoclaving	Thermo Scientific, Rockford, IL, USA
Three-repeat tau (RD3)	Mouse mono (8E6/C11)	1∶100	Autoclaving	Millipore, Temecula, CA, USA
Four-repeat tau (RD4)	Mouse mono (1E1/A6)	1∶100	Autoclaving	Millipore, Temecula, CA, USA
Tau (pan tau)	Rabbit poly	1∶100	Autoclaving	Sigma, St Louis, MO, USA
MAP2	Rabbit poly	1∶1000	Autoclaving	Millipore, Temecula, CA, USA
GFAP	Rabbit poly	1∶400	Autoclaving	Dako, Carpinteria, CA, USA
Olig2	Rabbit poly	1∶200	Autoclaving	Millipore, Temecula, CA, USA
GSK-3β	Rabbit poly	1∶100	Autoclaving	Cell Signaling, Danvers, MA, USA
Phospho-GSK-3β(Ser9)	Rabbit poly	1∶100	Autoclaving	Cell Signaling, Danvers, MA, USA
Ubiquitin	Rabbit poly	1∶200	Autoclaving	Dako, Carpinteria, CA, USA

PHF-tau, paired helical filament tau; MAP2, microtubule-associated protein; GFAP, glial fibrillary acidic protein; Olig2, oligodendrocyte transcription factor 2; GSK-3β, glycogen synthase kinase 3 beta.

## Materials and Methods

### Animal Brains

Most of the animals used in this study were wild animals that had lived exclusively on Tsushima Island, Nagasaki Prefecture, Japan. The Tsushima leopard cat is a subspecies of the leopard cat (*Prionailurus bengalensis*) [19]. The leopard cat was designated as a national endangered species in 1994 and ever since has been the focus of a conservation program funded by the Japanese government [http://kyushu.env.go.jp/twcc/multilang/english/pamph.htm].

A retrospective study was performed using paraffin-embedded tissues from 14 individual brains (Table 1). The brains were obtained from routine necropsies performed at the Laboratory of Veterinary Histopathology, Kagoshima University; Laboratory of Veterinary Pathology, Yamaguchi University; the Tsushima Wildlife Center of the Ministry of Environment of Japan; or the Department of Veterinary Pathology, the University of Tokyo. Most of these animals were killed in road accidents. No animal was killed for the purposes of this study. Unfortunately, the precise ages of the animals were not determined except for two individuals (Case No. 1∶3-days-old and Case No. 2∶3-years-old) that died at a reproduction facility (Table 1). Case No. 13 and 14 had been kept in captivity at a conservation facility for 10 and 15 years, respectively (Table 1).

**Figure 1:**
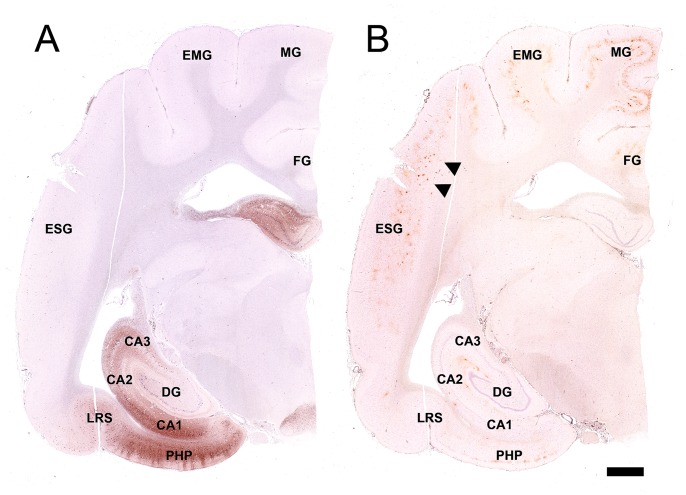
Distributions of hyperphosphorylated tau (A) and Aβ42 (B) in the leopard cat brains. (A) Hyperphosphorylated tau-positive cells were observed throughout the hippocampus and extended into the parahippocampal gyrus and the ectosylvian gyrus. (B) Aβ42 was deposited throughout the cerebral cortex as well as in the hippocampus. Speckled deposits of Aβ42 (arrowheads) were observed in a severely affected brain. Note that these deposits were not argyrophilic plaques. Bar = 2 mm. PHP: parahippocampal gyrus, DG: dentate gyrus, LRS: lateral rhinal sulcus, ESG: ectosylvian gyrus, EMG: ectomarginal gyrus, MG: marginal gyrus, FG: fornicatus gyrus.

### Histopathology

All brains were fixed in 10% phosphate-buffered formalin, coronally sliced, and then conventionally embedded in paraffin. The paraffin-embedded tissues were cut into 4-µm-thick serial sections. The deparaffinized sections were then stained with HE, periodic acid-methenamine silver (PAM), and the Gallyas-Braak method.

**Figure 2:**
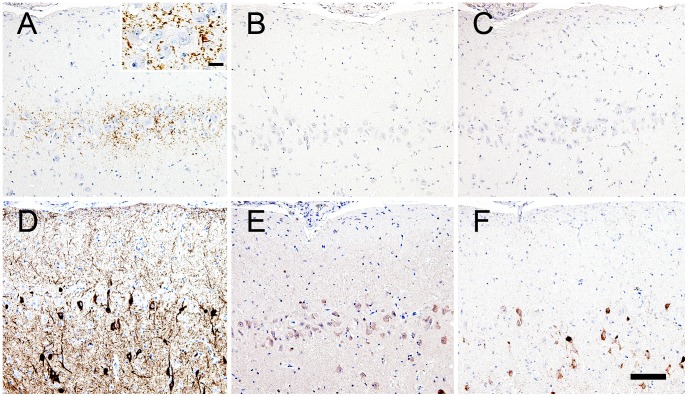
Sequential sections subjected to immunohistochemical examinations of Aβ and tau protein expression in the cerebral cortex.

### Immunohistochemistry

Consecutive sections were stained using the immunoenzyme technique. In order to deactivate endogenous peroxidase, the deparaffinized sections were immersed in 1% hydrogen peroxide in methanol for 20 minutes and then washed with Tris-buffered saline (TBS). The primary antibodies that were used in this study are listed in Table 2. After incubation with the primary antibody at 4°C overnight, immunolabeled antigens were visualized using the Dako Envision+ System (Dako, Carpinteria, CA, USA). In brief, the sections were incubated with HRP-labeled polymer at 37°C for 40 minutes, reacted with 0.05% 3′3-diaminobenzidine plus 0.03% hydrogen peroxide in Tris-hydrochloric acid buffer, and then counterstained with hematoxylin. Negative controls were obtained by omitting the primary antibodies.

**Figure 3:**
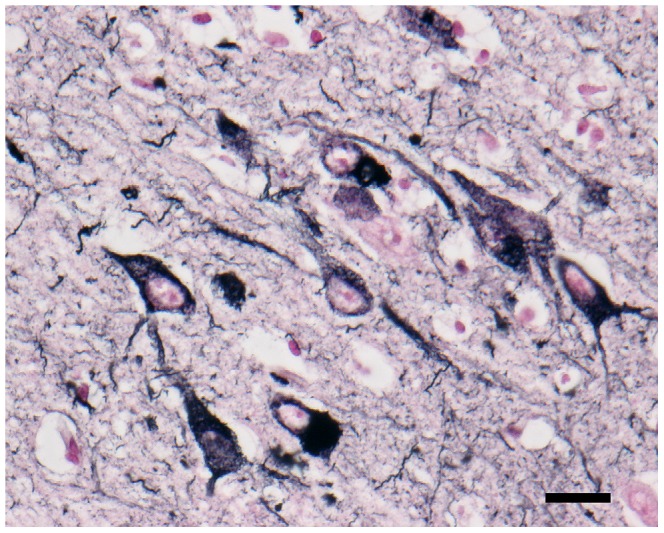
Gallyas-Braak staining. Argyrophilic NFT and neuropil threads were abundantly observed in the areas containing hyperphosphorylated tau-positive cells. Bar = 100 µm. Inset: higher magnification of the affected neurons. Bar = 20 µm.

### Indirect Double Immunofluorescence Staining

The double immunofluorescence staining technique was also performed to determine which cell types contained hyperphosphorylated tau. Anti-MAP2, GFAP, and Olig2 antibodies were used as markers of neuronal cells, astrocytes, and oligodendrocytes, respectively. In addition, the coexistence of hyperphosphorylated tau with glycogen synthase kinase 3-β (GSK 3-β), phosphorylated GSK 3-β (Ser9), and ubiquitin was also analyzed.

After incubation with the primary antibodies at 4°C overnight, the sections were washed with TBS. As secondary antibodies, ALEXA594-conjugated goat anti-mouse IgG (Invitrogen, OR, USA) and ALEXA488-conjugated goat anti-rabbit IgG (Molecular Probes, OR, USA) were mixed with TBS (dilution: 1∶100 for both antibodies). The sections were incubated with the secondary antibody mixture at 37°C for one hour, mounted with Vectashield (H-1500, Vector Laboratories, Burlingame, CA, USA), and examined under a Leica DMI 3000B fluorescence microscope (Leica Microsystems, Tokyo, Japan).

**Figure 4:**
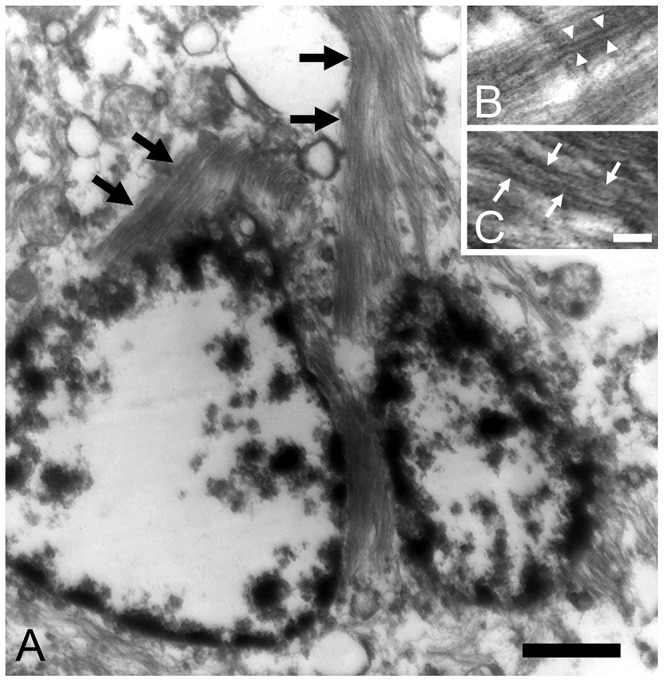
Electron micrographs of NFT. (A) Some neuronal somata and neurites were filled with filamentous bundles (black arrows). Bar = 1 µm. (B, C) The filaments formed paired structures with diameters of 10–20 nm. Straight laminar filaments (white arrowheads) and constrictions (white arrows) suggesting helical structure. Bar = 50 nm.

### Electron Microscopic Analysis

Formalin-fixed paraffin-embedded brain tissue from Case No. 13 was deparaffinized, cut into 1-mm cubes, fixed in 2.5% glutaraldehyde 0.1M phosphate buffer (pH 7.4), and then post-fixed in 1% osmium tetroxide 0.1 M cacodylate buffer (pH 7.2). The tissues were dehydrated in a graded series of ethanols, treated with QY-1 (Nisshin EM, Tokyo, Japan), and embedded in an epoxy resin (Quetol 651, Nisshin EM, Tokyo, Japan). Ultrathin sections from selected areas were stained with uranyl acetate and lead citrate and examined with a Hitachi H-7500 transmission electron microscope (Hitachi High-Technologies, Tokyo, Japan).

**Figure 5:**
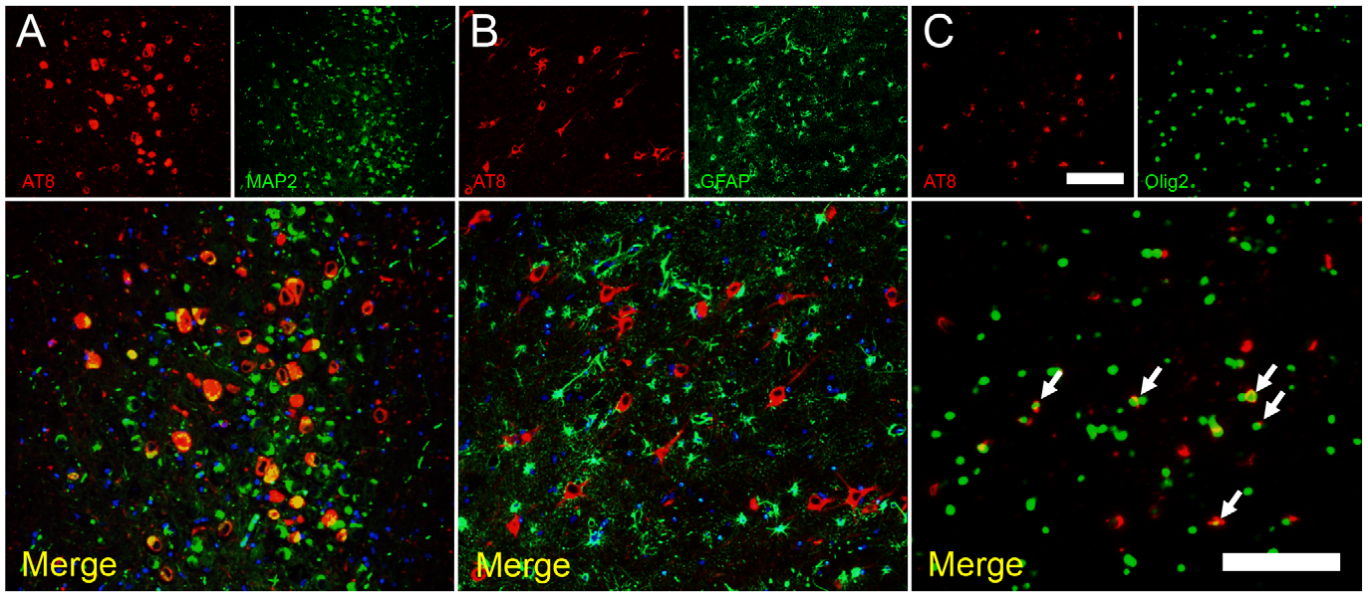
Double immunofluorescence staining of hyperphosphorylated tau (AT8)/MAP2 (A), AT8/GFAP (B), and AT8/Olig2 (C). Hyperphosphorylated tau was mainly localized in neuronal cells (MAP2+) and a few oligodendrocytes (Olig2+) (A, C), but not in astrocytes (GFAP+) (B). Bars = 100 µm.

### Scoring

The distributions of Aβ42 and hyperphosphorylated tau were assessed using the following scoring methods. Aβ42 deposition, −: none, +: diffuse Aβ42 deposition in the cerebral cortex, ++: diffuse Aβ42 deposition in the cerebral cortex and hippocampus, and +++: additional distinct plaque-like deposition; hyperphosphorylated tau, −: none, +: a few AT8-positive cells were found in the parahippocampal gyrus, ++: AT8-positive cells were found in the parahippocampal gyrus and hippocampal CA1 region, and +++: in addition to the parahippocampal gyrus and hippocampal CA1 region, AT8-positive cells were also found in the ectosylvian gyrus and hippocampal CA3 region.

**Figure 6:**
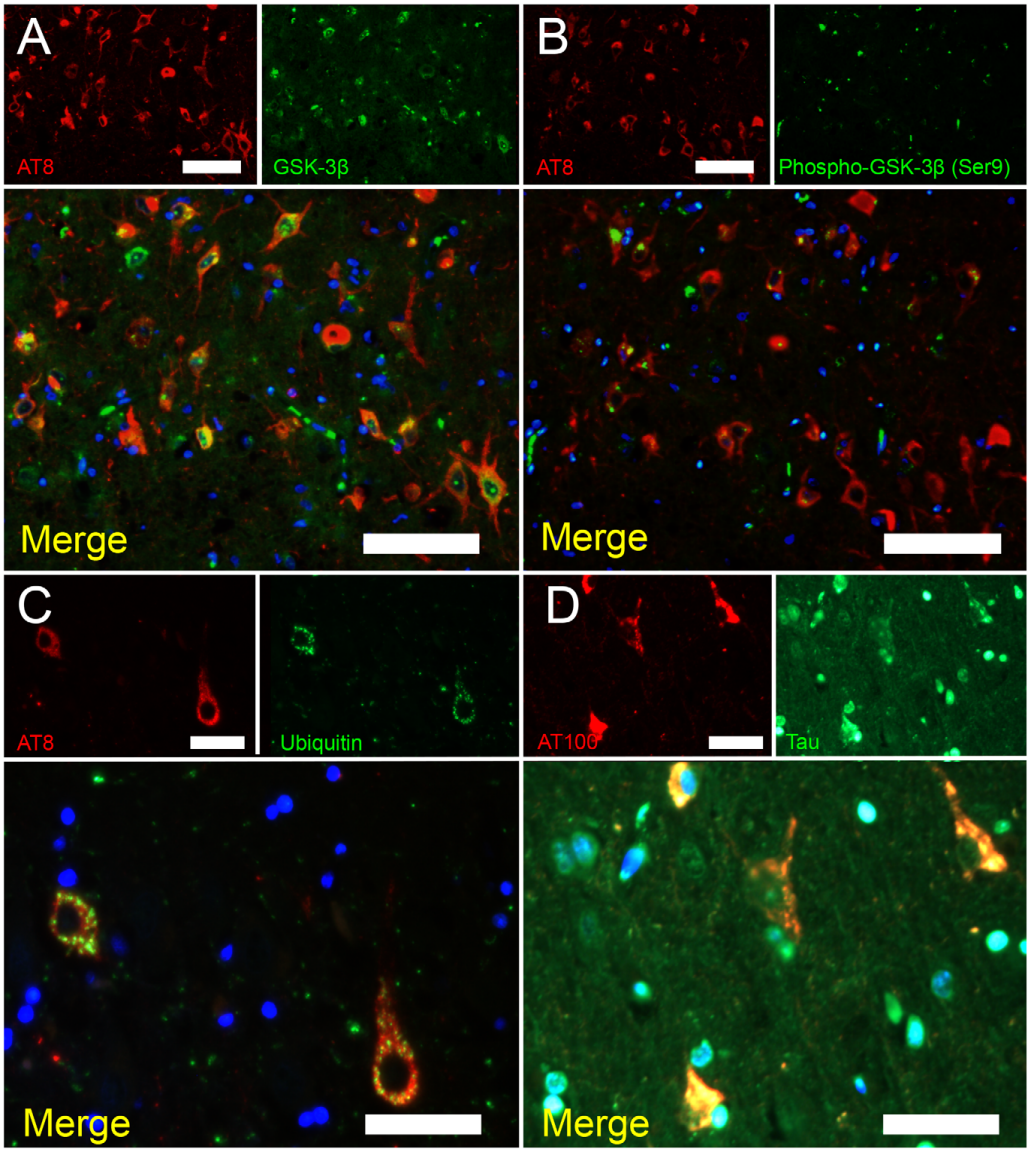
Double immunofluorescence staining of hyperphosphorylated tau (AT8)/GSK-3β (A), AT8/Phospho-GSK-3β (Ser9) (B), AT8/ubiquitin (C), and hyperphosphorylated tau (AT100)/tau (D). (A, B) AT8 colocalized with GSK-3β but not with phospho-GSK-3β (Ser9). Bars = 100µm. (C) Granular staining of ubiquitin was observed in the hyperphosphorylated tau-positive neurons. Bar = 50 µm. (D) AT100 colocalized with aggregated tau. Bar = 50 µm.

### APP Transcript Sequence Analysis

Total RNA was extracted from the formalin-fixed paraffin-embedded brain tissue of three leopard cats (Case No. 7, 8, and 14 were selected as they displayed the least postmortem changes) using the RNeasy FFPE kit (Qiagen, Tokyo, Japan). Subsequently, 10 ng of RNA were reverse-transcribed and amplified using the OneStep RT-PCR kit (Qiagen, Tokyo, Japan). For cDNA amplification, we designed a pair of primers covering exons 11–12 (forward primer 5′- AGATCCGGTCCCAGGTTATG-3′) and exons 16–17 (reverse primer 5′- GTCGACCTCCACGACACC-3′) of the domestic cat (*Felis catus*) APP gene (ENSFCAG00000001556). The PCR products were electrophoresed on 2% agarose gel and then purified using the QIAquick Gel Extraction kit (Qiagen, Tokyo, Japan). Direct DNA sequencing was accomplished using the BigDye Terminator v3.1 Cycle Sequencing Kit (Applied Biosystems, CA, USA) on the 3730×l DNA Analyzer (Applied Biosystems, CA, USA).

**Figure 7:**
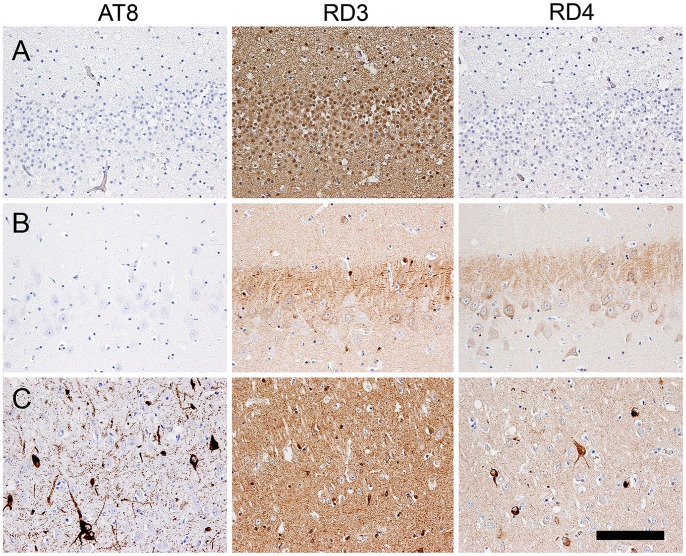
Sequential sections subjected to immunohistochemical staining of hyperphosphorylated tau (AT8), 3R-tau, and 4R-tau in the CA1 region of a neonate brain (A), an adult brain without AT8-positive aggregates (B), and an adult brain with AT8-positive aggregates (C). (A) Only the 3R-tau isoform was expressed in the brain of the neonatal leopard cat. (B, C) In the adult brains, both the 3R-tau and 4R-tau isoforms were expressed regardless of the presence or absence of AT8-positive aggregates. Bar = 100 µm.

## Results

In immunohistochemical examinations, the brains of 6 individuals were found to be positive for Aβ42, including 5 brains that were positive for hyperphosphorylated tau (Table 1). Note that all of the individuals that possessed hyperphosphorylated tau also displayed Aβ42 deposits, but the reverse was not true. The hyperphosphorylated tau-positive cells first appeared in the parahippocampal gyrus, and they subsequently spread through regions CA1 to CA3 of the hippocampus and into the ectosylvian gyrus (temporal lobe) in the more severely affected cases (Figure 1A). On the other hand, Aβ42 was initially diffusely deposited in the parietal and temporal cortices and subsequently spread to the hippocampal region. In some cases, Aβ42 was deposited in a speckled pattern (Figure 1B); however, these deposits were very diffuse, and none of these cases were stained with PAM staining. Also, Aβ42 was not deposited in the vascular walls.

**Figure 8:**
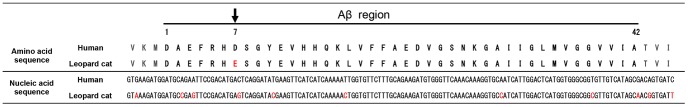
Nucleic acid and amino acid sequences of leopard cat and human Aβ region. In humans, the 7th amino acid residue of the Aβ peptide is aspartic acid (D), while in leopard cats it is glutamic acid (E).

In general, Aβ42 was granularly deposited in the neuropil of the pyramidal cell layer (Figure 2A). Interestingly, these deposits were not immunolabeled with anti-AβN1 antibody or anti-AβpN3 antibody on sequential sections (Figure 2B, 2C). In addition to Aβ peptides, aggregates of hyperphosphorylated tau were observed in neurites and perikarya (Figure 2D). These aggregates displayed intense staining for both the 3 repeat (3R) and 4 repeat (4R) tau isoforms (Figure 2E, 2F). Colocalization of pan tau antibody with hyperphosphorylated tau, 3R-tau and 4R-tau, also pan Aβ antibody with Aβ42 were confirmed (Figure S1). With Gallyas-Braak staining, argyrophilic NFT and neuropil threads were abundantly observed in the areas containing hyperphosphorylated tau-positive cells (Figure 3). Ultrastructurally, some neuronal somata and neurites had been filled with bundles of filaments (Figure 4A). These filaments formed paired structures with diameters of 10–20 nm. Most of the filaments were straight (Figure 4B), and some constrictions were also observed suggesting helical structure (Figure 4C).

Double immunofluorescence staining examinations revealed that aggregates of hyperphosphorylated tau had developed not only in neuronal cells but also in some oligodendrocytes (Figure 5A, 5C). Note that there were no astrocytic plaques (Figure 5B), which was also confirmed by the Gallyas-Braak method. In addition, the unphosphorylated form of GSK-3β, which is the major kinase involved in tau phosphorylation, colocalized with hyperphosphorylated tau in neuronal somata, whereas staining for phosphorylated-GSK-3β was negative in these cells (Figure 6A, 6B). The hyperphosphorylated tau-positive cells were markedly positive for ubiquitin, which was distributed in a granular pattern (Figure 6C). The aggregation of hyperphosphorylated tau was also confirmed by AT100 antibody (Figure 6D), which detects different phosphorylation site from AT8 antibody (AT8: Ser202/Thr205 and AT100: Ser212/Thr214).

**Table 3:** Senile plaque and neurofibrillary tangle formation in humans, leopard cats, and rodents.

Aβ protein (difference from human peptide)	Animals with identical Aβ amino acid sequences	SP formation	NFT formation	References
**Human Aβ**	Monkeys, Dogs, Bears	Argyrophilic plaques in aged individuals	Extremely rare, except in humans	[16], [17]
**Leopard cat Aβ** (1 amino acid residue)	Novel	Granular Aβ deposits, Noargyrophilic plaques	Often found in cases with Aβ deposits	Present study
**Rodent Aβ** (3 amino acid residues)	Rats, Mice	No reports of Aβ deposition	No reports of NFT	[18]

Both 3R-tau and 4R-tau were expressed in the brains of the adult individuals regardless of the presence or absence of NFT formation (Figure 7B, 7C). However, only the 3R-tau protein was expressed in the brain of the neonatal (3-days-old) leopard cat (Figure 7A).

Sequence analysis of the APP transcripts obtained from 3 leopard cats (Case No. 7, 8, and 14) revealed that their Aβ domains had identical sequences (Figure 8). Alignment with the human APP sequence (ENSG00000142192) showed 8 nucleic acid substitutions in the Aβ domain, one of which resulted in the substitution of the 7th amino acid residue (Asp in humans, Glu in leopard cats) of the Aβ peptide (Figure 8).

## Discussion

Many animal species develop Aβ deposits, especially higher mammals (e.g., monkeys, dogs, bears, camels, and horses) [4], [20], [21], [22], [23]. Most of these animals develop argyrophilic plaques, and some even develop mature plaques with amyloid cores. In contrast, felids seldom develop argyrophilic senile plaques, but granular aggregates of Aβ peptide are often observed in aged domestic cats and cheetahs [8], [11], [24], [25]. In the felid phylogenetic tree, the leopard cat lineage is located in between the cheetah lineage and the domestic cat lineage [14]. These three species are the most recent to have diverged among the 8 lineages of living felids. The findings obtained in this study further confirm the distinctive pattern of Aβ deposition that occurs in the brains of felids (Figure 2A). In addition, we found that the N-terminal epitope of the leopard cat Aβ peptide differs from that found in humans and other animals that develop argyrophilic plaques (Figure 2B, 2C, 8) [2], [16], [17], [26]. It has recently been established that the N-terminal subtype of Aβ peptides affects their aggregability, and hence, plaque formation [27], [28]. The alternative N-terminal epitope of the leopard cat might be responsible for the low aggregability of its Aβ peptides, which do not seem to produce argyrophilic plaques or vascular deposits.

Most interestingly, nearly all of the individuals that displayed Aβ deposition also possessed NFT (Table 1, Figure 3). Considering that non-human animals rarely develop NFT, a high incidence of NFT is likely to be a trait of this species. In order to determine whether these NFT correspond to the NFT found in AD, we investigated their histopathological characteristics. As we found that the distribution of hyperphosphorylated tau-positive cells in leopard cats was quite similar to that observed in human AD patients, we developed a scoring system based on the Braak staging method (Table 1) [29]. Although only a limited number of cases were available for study, subjective assessments suggested that the spread of tau hyperphosphorylation in leopard cats corresponds to the progression of AD [30].

Human diseases that involve the development of intracellular aggregates of tau protein, such as Pick’s disease (PiD), corticobasal degeneration (CBD), progressive supranuclear palsy (PSP), argyrophilic grain disease (AGD), and AD, are termed tauopathies. In CBD and PSP, tau often aggregates in astrocytic processes, forming lesions called astrocytic plaques and tufted astrocytes [31], [32]. PiD and AGD produce distinctive tau inclusions in neuronal cell bodies, which are known as Pick bodies and argyrophilic grains, respectively. In the brains of leopard cats, hyperphosphorylated tau aggregates in neuronal cells and some oligodendrocytes, but not in astrocytes (Figure 5). Oligodendrocytic tau inclusions are most prominently found in PSP and CBD, and to a lesser degree, in AD brains [33]. In sections of the leopard cat brains that had been stained with the Gallyas-Braak method, NFT and neuropil threads were observed (Figure 3), whereas no Pick bodies or argyrophilic grains were found. Ultrastructurally, the NFT were composed of paired filaments with diameters of 10–20 nm (Figure 4B, 4C), which is consistent with the properties of NFT in AD brains [34]. However, most of the filaments exhibited a straight laminar structure, rather than the helical structure that is often seen in AD brains [35].

Tau protein is associated with microtubules and promotes their polymerization and stabilization. Exon 10 of the tau gene encodes the second of four microtubule-binding repeat domains; therefore, the alternative splicing of exon 10 results in tau isoforms with either three (3R-tau) or four (4R-tau) microtubule-binding sites. Although the pathomechanism is yet to be elucidated, the dominant tau isoform in inclusions varies among diseases [36], [37]. PiD develops coiled filaments composed of 3R-tau alone [38], [39], whereas in CBD, PSP, and AGD the filamentous aggregates are composed of 4R-tau alone [40], [41]. In the brains of the leopard cats, both 3R-tau and 4R-tau aggregated in neuronal cells (Figure 2E, 2F, S1B, S1C) [36], [42], [43]. In addition, we found that only the 3R-tau isoform was expressed in the brain of the neonatal leopard cat (Fig. 7A). In human brains, tau expression shifts from the 3R-tau isoform alone to both the 3R-tau and 4R-tau isoforms between post-natal day 15 and 35 [44], [45], [46]. Therefore, the age-related expression pattern of tau isoforms and the components of the tau inclusions found in the leopard cat brains correspond to those of human aging.

GSK-3β is the major enzyme involved in tau phosphorylation, which culminates in NFT formation [47]. Phosphorylated-GSK 3-β (Ser9) is the inactive form of the enzyme, whereas the active form of GSK-3β (unphosphorylated) colocalizes with NFT (Fig. 6A) [48], [49]. In the amyloid cascade hypothesis of AD, GSK 3-β links Aβ deposition and tau hyperphosphorylation in the pathological sequence [50].

As mentioned above, animals that develop argyrophilic senile plaques, such as monkeys and dogs, are known to display the same Aβ peptide amino acid sequence as humans. On the other hand, Aβ deposition has never been demonstrated in non-transgenic wild-type rodents, such as rats and mice. Rodent Aβ displays three amino acid differences in its N-terminal region compared with human Aβ, which are presumed to account for the absence of amyloid deposits in wild-type rodents [30]. As it was also the case in the leopard cat, the different amino acid residues are intensively located in the N-terminal region of the Aβ protein [18]. The findings of the present study indicate that the leopard cat Aβ peptide has an intermediate aggregative nature between those of human Aβ and rodent Aβ (Table 3). There is increasing evidence to suggest that weakly aggregative forms of Aβ are more important for neurodegeneration than classical argyrophilic plaques consisting of a mass of aggregated Aβ protein [51], [52], [53], [54], [55], [56]. However, wild-type rodents with non-aggregative Aβ do not develop NFT. In a study using PS1×APP transgenic mice, the age-dependent accumulation of small Aβ aggregates was found to be related to decreased GSK-3β phosphorylation, which resulted in tau phosphorylation [57]. Since AD-type NFT have never been observed in the brains of monkeys or dogs with senile plaques, it is generally considered that such non-human animals die before NFT develop [13]. The leopard cats that were examined in this study are only found on Tsushima Island, Japan. This subspecies has been geographically isolated on this island for approximately 0.1 million years, and a 2005 survey estimated that only 80–110 Tsushima leopard cats remain [http://kyushu.env.go.jp/twcc/multilang/english/pamph.htm]. The lack of genetic diversity in this subspecies should be taken into consideration as a potential factor in the peculiar AD pathology seen in these animals.

## Supporting Information

Figure S1 **Double immunofluorescence staining of tau/hyperphosphorylated tau (AT100) (A), tau/3R-tau (B), tau/4R-tau (C), and Aβ/Aβ42.** (A, B, C) Hyperphosphorylated tau, 3R-tau and 4R tau colocalized with pan tau antibody-positive aggregates. Bar = 20 µm. (D) Granular staining of Aβ42 colocalized with pan Aβ antibody. Bar = 100 µm.
(TIF)
