# Supplementary figures and images for: Neurofibrillary Tangles and the Deposition of a Beta Amyloid Peptide with a Novel N-Terminal Epitope in the Brains of Wild Tsushima Leopard Cats
Source: PLoS One. 2012 Oct 3;7(10):e46452. doi: 10.1371/journal.pone.0046452 (PMC3463583; doi:10.1371/journal.pone.0046452)

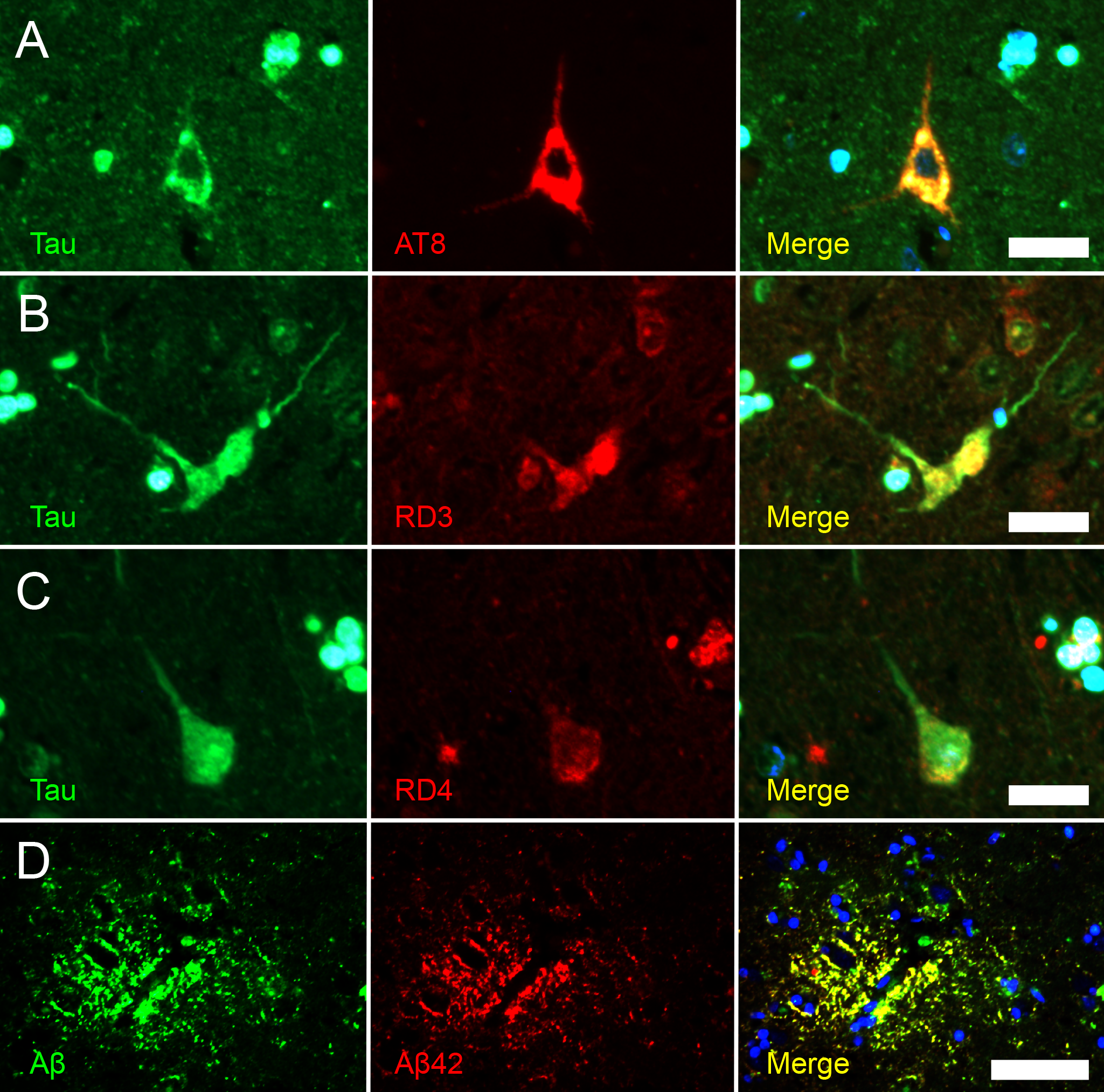

Supplement: Figure S1 — Double immunofluorescence staining of tau/hyperphosphorylated tau (AT100) (A), tau/3R-tau (B), tau/4R-tau (C), and Aβ/Aβ42. (A, B, C) Hyperphosphorylated tau, 3R-tau and 4R tau colocalized with pan tau antibody-positive aggregates. Bar = 20 µm. (D) Granular staining of Aβ42 colocalized with pan Aβ antibody. Bar = 100 µm. (TIF) [file pone.0046452.s001.tif]
